# Parent-child communication about substance use, puberty, sex, and social media use among Hispanic parents and pre-adolescent children

**DOI:** 10.1371/journal.pone.0295303

**Published:** 2023-11-30

**Authors:** Yui Matsuda, Roxana D. Thalasinos, Alexa Parra, Roberto Roman Laporte, Maria A. Mejia-Botero, Abgail L. Adera, Melody Siles, Gerardo Lazaro, Ronak N. Venkata, Joseph P. De Santis

**Affiliations:** School of Nursing and Health Studies, University of Miami, Coral Gables, FL, United States of America; Florida Atlantic University Charles E Schmidt College of Medicine, UNITED STATES

## Abstract

**Background & purpose:**

Previous research has noted that Hispanic pre-adolescents may be at an increased probability for engagement in risk-taking behaviors. The purpose of this study was to explore parent-child communication among Hispanic parents and 4th-6th grade children related to substance use, puberty, sex, and social media use.

**Methods:**

A qualitative descriptive design was used to examine Hispanic parents’/caregivers’ communication with their children about substance use behaviors, pubertal developments, engagement in sexual risk behaviors, and social media use. The study included two components: four focus groups consisting of 23 children; five focus groups and one interview consisting of 24 adults. All were conducted until data saturation was reached. Parents and pre-adolescents were interviewed separately. Interviews with parents and pre-adolescents were audio-recorded, transcribed verbatim, and analyzed using content analysis techniques.

**Results & conclusion:**

The themes that emerged from the interviews were about children’s feelings, parents’ feelings, communication messages that children received from their parents, and information parents provided to their children during parent-child communication. The results indicate discrepancies between information that parents provided and information that the pre-adolescents reported. The results have implications for healthcare providers in that parents need to be better educated on communicating effectively with their pre-adolescents about risk-taking behaviors. Healthcare providers may help facilitate parent-child communication with Hispanic families. More research is needed to develop intervention programs for Hispanic parents to learn how to effectively communicate with their pre-adolescent children in a developmentally appropriate manner.

## Introduction

Pre-adolescence, defined as the period of human development right before adolescence, is a crucial stage of child development [1]. During this period, children experience physical and emotional changes, and peer pressure increases [2]. A solid psychological and emotional foundation built during pre-adolescence will lead to healthy adolescence and young adulthood [3]. Even though risk-taking behaviors such as drinking, substance abuse, and sex tend to become more prevalent during adolescence, these behaviors are preventable and can be addressed through early interventions to mitigate the potentially negative consequences. Studies have shown various negative impacts of risk-taking behaviors; the use of substances such as alcohol has been shown to lead to structural brain changes, decreases in brain matter, and possible neural impairments [4]. Substance abuse, whether it be alcohol, tobacco, or marijuana, have all been linked to suicidality in varying degrees [5]. Risk-taking sexual behaviors pose additional potential problems, including increases in teen pregnancies and sexually transmitted infection (STI) and/or HIV infection rates, especially when combined with other risk-taking behaviors. All of these behaviors in tandem can lead to increases in emotional stressors and financial burdens and can affect all aspects of adolescents’ lives [6]. Social media use also allows pre-adolescents and adolescents to be exposed to content promoting early engagement with substance use and sexual activity [7]. For example, a recent meta-analysis of 27 independent cross-sectional studies has confirmed previous findings that social media use positively correlates with risk-taking behaviors such as substance abuse and high-risk sexual behaviors among adolescents [8]. As there is evidence that pre-adolescents are using social media at equal or higher rates to that of their adolescent counterparts [9], it is clear that social media use is an important topic in parent-child communication to mitigate risk-taking behaviors among this age group.

Hispanic youth are a population of concern for these risk-taking behaviors: These youth have the highest prevalence of drug use (synthetic marijuana, cocaine, and crack; [10] and a significantly high rate of vaping compared to other races/ethnicity [11]. Regarding sexual behavior, Hispanic youth have lower rates of contraceptive use and higher rates of pregnancy than other ethnicities [12]. To add to the to sexual health risk, Hispanic adolescents who reported higher frequency of social media use had greater odds of engaging in some form of sexual contact [13]. These statistics present findings from Hispanic adolescents (13 and older) because the national survey studies only collect data on adolescents’ risk-taking behaviors. However, considering the associations between social media use and risk-taking behaviors among adolescents [8], prior research suggests that there is an urgent need to prevent and reduce risk-taking behavior among children before they reach adolescence. Recent literature suggests that a parent-centered approach may reduce the prevalence of risk-taking behaviors among youths [14]. Evidence shows that the effectiveness of parent-child communication can strengthen the relationship between parent and child [2]. This is important because the presence and involvement of parents plays a positive role for pre-adolescents and adolescents, influencing peer relationships, academics, physical and emotional changes, and well-being [2, 15]. Conversely, the absence of parental involvement in the child’s activities and poor parent-child communication leads to a higher prevalence of risk-taking behaviors [16]. One significant complicating factor is that Hispanic parents rarely speak to their children about sexual matters, and when they do, it is usually after the sexual behavior has started [17].

Although previous research has found that improving parent-child communication is associated with fewer risk-taking behaviors (e.g., alcohol, tobacco, and sex) [18, 19], these studies have tended to target parents and adolescents, not pre-adolescents. Thus, introducing a preventative intervention focused on Hispanic pre-adolescents can subsequently improve the reduction of substance use and risk-taking sexual behavior in adolescents [20]. Furthermore, Guilamo-Ramos et al. [21] discussed the importance of parents as a primary source of health information for pre-adolescents. However, there are currently no parent-based interventions targeting Hispanic pre-adolescents to promote parent-child communication about risk-taking behaviors related to both substance use and sex. This study explored parent-child communication among Hispanic parents and 4th-6th grade children related to substances, puberty, sex, and social media use so that we can understand how to address these important health topics.

## Methods

### Study design

The study used a qualitative descriptive design. This design describes the phenomena of interest from the data by applying a naturalistic paradigm [22, 23]. Focus groups were conducted separately with parents and children to facilitate in-depth exploration of each group. The University of Miami’s Institutional Review Board approved all procedures used in this study. Adult participants gave written informed consent for themselves and their child(ren). Additionally, pre-adolescent participants gave written assent to participate in the study.

### Participants and procedure

Participants were recruited from an afterschool program run by a local community-based organization which serves mainly Black and Hispanic families. Inclusion criteria of children were: (1) in the 4^th^-6^th^ grades; (2) of Hispanic origin; and (3) spoke English. English was observed to be the preferred language for the after-school program children to express their thoughts and emotions more freely. This is reasonable when considering that the children were mostly born in the U.S. or lived most of their lives in the U.S., and attended schools in which English is the language of instruction and the dominant language for peer socialization. Inclusion criteria of parent/primary caregiver/legal guardian included: (1) parent/primary caregiver/legal guardian (referred to as “parents” from now on) of a child who is in 4^th^-6^th^ grade; (2) of Hispanic origin; and (3) spoke English or Spanish. The principal investigator (PI; Y.M.), bilingual in Spanish and English and bicultural, met with 28 parents in-person when they came to pick up their children at the afterschool program. In a private location, the research staff met with each eligible parent to explain the study and the written consent forms in his or her preferred language; parents consented to their own participation in their focus group and their children’s participation, respectively. The PI ensured that all the parents’ questions were answered before obtaining consent. The study personnel emphasized that study participation is voluntary and that not participating in the study does not affect the services their children receive at the afterschool program. Of the 28 parents, 2 declined to participate in the parent focus group because of irregular work schedule (*n* = 1) or not feeling comfortable to participate (*n* = 1). However, both consented for their children to participate in the study. Of 26 adults who consented for the parent focus groups, two did not show; thus, 24 parents participated. Each parent participant attended one in-person focus group at a time of convenience, either in the morning or evening. For the children’s focus groups, trained study personnel explained the study to children using the assent form. Children’s focus groups were conducted while children were in the afterschool program. The study personnel ensured that all children’s questions were answered before the children signed the form. The study personnel informed children that if they do not wish to participate, they could return to their activity. A total of 23 children participated in the study (See Table 1 for parent demographics and Table 2 for child demographics).

**Table 1:** Parent demographics(*n* = 24).

Variable	*n* (%)
Gender	
Male	1 (4.2)
Female	22 (91.7)
Prefer not to answer	1 (4.2)
**Place of birth**	
Mexico	10 (41.7)
El Salvador	6 (25)
Cuba	2 (8.3)
Honduras	2 (8.3)
Puerto Rico	2 (8.3)
Nicaragua	1 (4.2)
Continental US	1 (4.2)
**Education completed**	
None	1 (4.2)
Primary school	9 (37.5)
Middle school	5 (20.8)
High school	6 (25)
Technical/Vocational	1 (4.2)
University or Post-Graduate	2 (8.4)
**Household income (annually)**	
Less than $10,000	5 (20.8)
$10,000 - $29,999	8 (33.3)
$30,000 - $49,000	3 (12.5)
$50,000 - $74,999	2 (8.3)
$75,000 - $99,999	2 (8.3)
Prefer not to answer	4 (16.7)
**Age** [*M*, (SD)]	38.9 (6.51)

**Table 2:** Child demographics (*n* = 23).

Variable	*n* (%)
**Gender**	
Male	7 (30.4)
Female	15 (65.2)
Prefer not to answer	1 (4.3)
**Puberty-related changes**	
Axillary hair	4 (21.7)
Started menstruation^a^	3 (20.0)
**Place of birth**	
US-born	21 (91.3)
Non-US born	2 (8.7)
**Age** [*M*, (SD)]	10.8 (1.01)

^a^The percentage of ‘started menstruation’ is based on *n* = 15 because this

category only applies to females.

### Procedures

All research team members completed role specific orientation and related training sessions prior to the data collection. For children, there were four focus groups with 4–7 participants in each group (group 1: *n* = 6; group 2: *n* = 4; group 3: *n* = 6; group 4: *n* = 7). For parents, there were five Spanish focus groups with 2–9 participants in each group (group 1: *n* = 2; group 2: *n* = 5; group 3: *n* = 3; group 4: *n* = 5; group 6: *n* = 9) and 1 interview in English (group 5; the participant preferred English). Focus groups/interview were conducted in March 2021. The focus groups took place in a privately designated room at the after-school program facility.

The focus groups/interview each lasted approximately one hour and were audio-recorded. Participants were compensated ($40 cash for parents; $20 gift card for children). The PI, a PhD prepared nurse, and a research support coordinator who has worked with improving Hispanic family health for over a decade (R.D.T.) created a semi-structured focus group guide [24]. Questions asked included: “What makes it (uncomfortable/comfortable) to talk about (alcohol/smoking cigarettes/vaping/drug use/sex and contraception)?” Probing questions were then asked by the facilitators to further explore the discussions. The parent and child focus groups used similarly structured outlines based on the same types of questions. The research support coordinator, a bilingual (Spanish and English) and bicultural person, conducted all of the parent focus groups in Spanish. A research assistant (R.R.L., a current PhD student with a Doctorate of Nursing Practice) conducted the parent interview and half of the children’s focus groups in English. The PI conducted the other half of the children’s focus groups in English. All of the focus group facilitators have facilitated or moderated focus group discussions prior to this study. A co-facilitator and/or notetaker (an undergraduate Research Assistant [RA]) was present for each focus group but not for the interview). One individual interview was conducted because this one participant preferred that the interview was done in English.

### Data analysis

The study used a qualitative descriptive design. This design is used to describe the phenomena of interest from the data by applying a naturalistic paradigm [22, 23, 25]. Content analysis was used to analyze the focus groups/interview data [26]. First, conventional content analysis was conducted to identify major themes emerging from the children’s focus groups. Conventional content analysis is an inductive method to capture the essence of the data without preconceived notions [25]. Second, a combination of directed and conventional content analysis was used to identify major themes emerging from the parent’s focus groups/interview. Data analysis began with directed content analysis (applying pre-set codes) [25] by using themes that emerged from the children’s focus group data to inform the parents’ focus group data analysis. To eliminate as much bias as possible, the research team incorporated the essence of conventional content analysis for any additional themes specific to the parents’ data.

The audio-recorded focus group discussions/interview were transcribed verbatim by trained and qualified RAs (e.g., Spanish focus groups by bilingual RAs). At least one other research team member who did not participate in the original transcription reviewed the transcriptions to improve accuracy. For the children’s focus groups, the PI and a research assistant (R.R.L.) conducted the data analysis. Both coders had prior experience in conducting qualitative data analyses. For the parents’ focus groups/interview, two Hispanic RAs who are bilingual in Spanish and English conducted data analysis, a PhD student in nursing (A.P.) and an undergraduate student majoring in public health (M.M.B.).

For the data analysis procedure, all of the coders were trained to conduct qualitative data coding and in the use of the NVivo software prior to starting their work. The team first worked on analysis of the children’s data: The two coders (Y.M., and R.R.L.) read and organized focus group/interview transcripts per topic area and identified emerging themes. Another research team member of Hispanic origin, a postdoctoral fellow in public health (G.L.), also joined these meetings to provide insight. These three team members met remotely several times to discuss emerging themes and create the codebook using an iterative process [27]. Based on the codebook definitions, coders analyzed the data while meeting regularly to discuss progress and challenges. The codebook further evolved based on these discussions. The coders’ interrater reliability was substantial (*k* = 0.72) [28]. The discrepancies were resolved before finalizing the coding.

For the parents’ data, the PI, who has experience in qualitative data analysis, regularly met with the coders to guide the process. Similar to child focus groups, the two coders (A.P. and M.M.B.) first read and organized the focus group/interview transcripts in their original language per topic area. Then, the coders discussed the relevance of the codes generated from the child focus groups for the parent groups/interview and revised phrasing as needed. Based on the codebook created, coders analyzed the data while meeting regularly to discuss challenges and ask questions. The coders’ interrater reliability was moderate (*k* = 0.46) [28]. The coders discussed discrepancies, reached a consensus, and finalized the coding process. The data analysis was led by the PI of the study (YM), who is an experienced qualitative researcher. The PI held weekly meetings with the research assistants conducting data analysis to discuss the findings and interpretations, as well as to examine the process and the results of the research study.

Through data saturation, the analysis continues until no new themes emerge, no new information is obtained, and redundancy is achieved [29]. No more focus groups were conducted once data saturation was reached. This means that focus group facilitators heard the same themes and did not gain new information after a certain number of focus group sessions. The children had four focus groups with 4–7 participants in each group. For parents, there were five Spanish focus groups with 2–9 participants in each group and one interview with a single parent.

The dependability of this study was confirmed through external audits. External audits involved having a researcher that is not a member of the data analysis examine both the process and result of the research study [30]. To achieve dependability using external audits, this study employed a graduate research assistant and a postdoctoral fellow who were not involved with the study proposal, design, data collection, or analysis to conduct the audit. The purpose of using this technique is to evaluate whether the findings and interpretations were supported by the data [30].

Additionally, to ensure credibility, peer debriefing was conducted during weekly team meetings with the data coders and PI. This was an opportunity for the research team involved with data analysis to uncover perspectives, biases, or assumptions made on the researchers’ part [30]. Furthermore, the research team continued to establish credibility through member checking with the leaders of a community-based organization from which participants were recruited. The PI and members of the research team met with them to summarize the main findings of the study and give them an opportunity to assess the adequacy of the data and results [30].

Confirmability was achieved through triangulation and audit trails. Through the triangulation of data, the research team collected data using different sources, methods, and investigators [27]. This study used multiple analysts to review findings to understand the multiple ways one can see the data and illuminate biases in an interpretation [31, 32]. The focus groups and the interview were audio-recorded and transcribed verbatim for analysis. Additionally, field notes were written by the focus group facilitators, co-facilitators, and notetakers, providing multiple sources and methods to understand contextual and non-verbal cues observed during the data collection. Audit trails also were conducted by using raw data, which included focus group transcriptions and written field notes. The data reconstruction and synthesis products [30] can be verified in the participant quotations, a codebook, and a code sheet.

## Results

The themes that emerged from the focus groups/interview are categorized as follows: 1) Children’s feelings, 2) parents’ feelings, 3) what children heard from parents, and 4) what parents told children. Themes 3 and 4 contain sub-themes to further categorize what children heard from parents and what parents told children.

### Children’s feelings

This theme highlights how serious conversations (e.g., puberty, drugs, sex) made children feel. Participants stated they felt “grossed out” and “awkward” discussing feelings about a boy or girl, puberty, and sex. One participant stated, “Yeah. I like felt grossed out about some of the stuff in our body parts and like how much we are growing […] and a lot of stuff that goes on inside of our body” (FG1). Some participants who have not had conversations about puberty and sex stated that they would feel “embarrassed” or “awkward” if parents were to speak about these topics. Some children believed they were too young to discuss puberty and sex and believed “that’s for later in life” (FG4). Additionally, as awkward and embarrassing conversations about puberty and sex could be, the majority of the children still preferred to have the conversation with their own parents and not with other people such as friends or relatives. One participant stated, “I would rather my parents saying it to me” (FG1).

On the other hand, topics such as substances (e.g., alcohol, cigarettes, and other drugs) elicited other feelings. Children felt that discussing cigarettes was “pretty normal” (FG1), but felt uncomfortable when parents thought their children smoked cigarettes or e-cigarettes. One participant stated, “they [parents] thought I did it [smoked e-cigarettes], but I am like underage and they [parents] didn’t understand” (FG1). Children also felt more uncomfortable discussing illegal substances such as marijuana: “it felt kind of weird…” (FG1); and “I just don’t feel comfortable enough to talk [about marijuana]” (FG 4).

### Parents’ feelings

Some parents at first felt uncomfortable discussing the topic of their children liking a boy or girl. A parent said, “In the beginning, there were times like I was little afraid to ask about that, but as she got older, yeah, yes” (FG 4). However, many parents spoke about feeling worried or expressed difficulty accepting their children liking people of the same sex. Some expressed desires to engage with the child and emotionally connect with him or her, while others expressed worries about the conflict they feel against their beliefs. A parent stated, “…you have to have… a lot of open mind and a lot of preparation to, not to tell them ‘No, it’s wrong,’ but to know how to guide them” (FG1). Another parent stated, “…God made man, woman, I wouldn’t like that she likes a girl, even though I know I need to accept my children the way they are…this is the topic that we (my daughter and I) talk about a lot” (FG2). Parents were also worried that their children were influenced by social media that normalize same sex relationships or choosing one’s gender.

Puberty and its physical changes were also discussed as an important topic or one raised by children. A parent stated, “Say…she starts saying, ‘look, you gonna see hairs’ then say, ‘Ah, oh yes, that’s fine’ and left like (my child) wasn’t interested in the topic. So, I stopped there” (FG 3). Some found it difficult to speak with their children about puberty-related changes in an easy-to-understand manner. A parent stated, “you know when the puberty and adolescence comes, I think that’s a very difficult part. Uh… in education, what are you going to teach her or… or… I don’t know. To have one as an example… (FG1)”

As far as sex, some parents said they have spoken with their children about this topic. One parent felt strongly about avoiding the same mistake they made by initiating sex at an early age, saying,

I said, “Look at your mother, I explained it back to my… my life… I made mistakes. I wasn’t man enough to deal with it…” But I try to explain to them I said, I said,”Your mom struggled…Yeah, we made mistakes. Don’t do it. Wait till you’re old enough.” Like I said,”Wait till you’ve got your goals set, your priorities right.” (FG5)

Some began to engage in these conversations as children started asking questions, which caught the parents by surprise. Nevertheless, some parents were able to address children’s questions. A parent said,

In this case it went from zero to ten, because no, I had no idea what, what she was gonna ask me at that moment. And it turns out that she comes and said, ‘you know what? I talked to such and such, a friend of hers, and she talked to me about sex.’ I mean, imagine you’re cooking and they tell you they talked about sex. Right there, I stopped the cooking and I told her ‘let’s talk about sex. What do you know?’ (FG4)

Although talking about sex required sensitivity and carefulness, some parents were able to manage the conversation. The majority of parents felt it was important to talk about sex, but did not know how to do so. A parent stated, “Very difficult. Sometimes even embarrassed [laughs]…but yes one has to talk…" (FG1).

Conversely, talking with their children about alcohol, cigarettes/e-cigarettes, and drugs/illegal substances was not something parents initially associated with feelings of embarrassment or difficulty as it did in talking about sex; however, a parent felt ambivalent as her child was young at the time of conversation. She stated,

Well, my husband told them, yes, they are going to face this many times, say now that they enter a middle school,…there will be more offers. He said, ‘you just stay far from these people, whenever you see them, even though they are your friend…your classmate, stay away from these people and (say) no.’ Well, this was a small conversation, but…I think that…my son is still small, 10 years old… (FG4)

Speaking with their children about cigarettes/e-cigarettes, a parent spoke about the shock she felt when her child was accused of being involved in possession of a vaping device, even though the child did not know what the device was for. She said, “vaping at this age?…it was a shock and a learning experience at that moment… it is very important because things have changed and they are not the typical topics that we used to talk about in our time…” (FG4).

### What children heard from parents

#### “Don’t Do XYZ” (or “Don’t do this/Don’t do that”)

Children stated that their parents spoke with them about different topic areas. Parents used “don’t do this” or “avoid that” type of language with little or no explanation. For example, when asked about parental advice regarding saying no to offers and invitations, a participant said, “staying away from bad friend” (FG1). Another mentioned, “Don’t hang around people that do it, it’s a bad influence” (FG4). When speaking about what parents said about smoking cigarettes/e-cigarettes, a participant commented, “To not do it…To never hang around people that do it” (FG4). Some participants indicated that their parents added a little about why they should avoid certain things/people. In reference to smoking cigarettes/e-cigarettes, a participant explained that “They just say it’s bad for you. Don’t do it…Yeah, also they told me that never be with wrong people because they could be a bad influence and they could make you do that, too” (FG4). Another participant said, “They told me not to do that, like… that it could hurt your lungs, yeah” (FG3).

#### Changes with your body

Participants stated that their parents spoke with them about puberty. A participant said, “It’s just, it’s just…it’s just like, like what’s going on in your body like they talk about what are just some stuff that is happened about in your body. That’s it” (FG4). Additionally, a female participant discussed what her mother said in regards to menstruation:

I just wanted to say that my mom always talks about those things with me. And she always says like, “Oh if this happens, if this [menstrual blood] comes out or whatever, like, um, like, she always tells me where they’re [pads/tampons] at, just in case, if I need.” I don’t know if you understand? (FG3).

A male participant spoke about his experience: “…we talked about…puberty. My dad talked about, like, what they’re gonna go through. Don’t be afraid, just calm down” (FG3).

#### Related to sex

There were only few specific comments about sex talk. A participant commented that her mother showed her a video explaining how one gets pregnant:

…sometimes they tell me about family friend and then someone sends them a video and then they just and then the person’s like, “Oh show this to um, your kid so they know how people get pregnant so they know how babies come out” and then when my mom calls me and like says like she she first tells because she doesn’t want to shows me things that and then I’m like noo? Because umm… it’s disgusting (FG2).

In contrast with this example, several participants stated that their parents only joke about how they were born or where babies come from. A participant said, “They joke about it…like a bird brought me from the sky or something like that” (FG3). Another participant said, “They just say one sentence. ‘Don’t do it when you’re young’” (FG3).

#### Social media

Some participants spoke about the danger of social media/internet platforms. A participant reported that parents said, “Don’t friend anybody” (FG3). Participants also said that their parents warned them about people they may encounter on social media. A participant mentioned, “Pedophilia” (FG4). Another said that “My auntie and my mom and my cousin says that Tiktok are Chinese, China made the app and they can see your emails” (FG 4). Another participant also commented about Tiktok: “Yes, my mom said that to be careful because sometimes they know where is your house, and they take you” (FG4). Moreover, participants mentioned that their parents spoke about appropriate conduct related to social media. A participant stated, “not to say the bad words to other people, and like, don’t say nasty stuff” (FG3).

### What parents told children

#### Warning

Parents said they used different words to express what children should or should not do when facing potentially risk-taking situations. Related to liking boys, a parent stated, “don’t do anything you’re not supposed to do” (FG5). Moreover, a mother emphasized the importance of being careful in relation to getting pregnant: “Well, on my end, like she is a girl, I always tell her that she has to protect herself a lot like thinking of her age… when her (period) start, I start talking about it” (FG4).

In relation to saying no to offers/invitations, a mother stated, “No, yes, always. I tell her that no, that no, that that is bad, that she can’t be doing that ever…” (FG1). Another parent stated, “Yes. I tell them that when they are invited to something that they should tell the person inviting that ‘no and thank you’” (FG4). Additionally, parents told their children to be careful as there are people out in the world who try to kidnap children. This applies both to when they are outside and on social media or the internet. A mother stated how her daughter needs to be careful even when they are at a park. She said,

Yes, that these days girls are hurt, that when sometimes we are walking or something. Eh, like yesterday we went to the park, she (child) wanted to go alone to ride the bicycle. I told her, no, because you don’t know who in this park is watching to sexually abuse you, to touch a child. I’ve always told her that (FG2).

A parent also talked about being careful regarding posting on social media:

We try and explain to them… There’s nothin’ wrong with you wanting to post a picture but just be careful. I said, you can see it for yourself on TV, the news, […] people, that’s how a lot of people getting killed or kidnapped. Social media! They find where you went, it could be a 50, 60 year old man. He wants you, he gonna come and get you. He don’t care. He ain’t got nothin’ to lose. You got your whole life ahead of you. But… they know, they not very cautious with things themselves (FG5).

#### Puberty

Parents stated that they told children that puberty is something that occurs in their body, mostly related to physical changes. A parent stated, “I mostly say, dear, take a pad, every day, I say that (laughs). She and I are traumatized…” (FG6). Another parent said, “Yes, we’ve had the talk, for example, they’re going to, um, use deodorants, I told him/her we’re going to start using deodorants because now we are starting to smell more” (FG3).

#### Ask me!

Parents said they told their children to talk with them instead of talking with their friends or using the internet when they have questions about sex and other topics about which the children may be hesitant to ask. A mother stated,

Considering that, the best you can hear the information it’s going to be from me than something erroneous from a friend, or who knows, whatever downloaded from the internet… I explain to her that internet has no filter, it’s very open and that I’d like her to tell me everything she wants to know, even though I am not an encyclopedia but yes obviously, I have more experience than her…Whatever you have questions about, before asking a, a friend of yours, as you always do, ask me… (FG4).

#### Stay in tune with children

This theme refers not to what parents said but to how they approached speaking and adding context to the topics. Parents spoke about being sensitive to cues and questions so they can address topics of interest when children are engaged. A mother stated, “So, I am like eh…eh watching out, no? depending on the topic that comes up, but then say ‘ok, come. you said such, let’s talk about it.’” (FG4) This mother further described how her child watched videos about drugs and started asking questions. Because she was aware of this topic, she was able to address the child’s questions using an open discussion.

## Discussion

The purpose of this study was to explore parent-child communication related to substance use, puberty, sex, and social media use among pre-adolescent (4^th^ to 6^th^ grade) Hispanic children and their parents in (*City*, *State; blinded for peer review*). This study is the first to describe parent-child communication about risk-taking behaviors among Hispanic parents and their pre-adolescent children.

Findings from our study support the idea that while many of the pre-adolescent children felt “awkward” discussing topics such as puberty and sex, they preferred these conversations be with their parents instead of other adults, which is consistent with existing literature on parent-child communication among adolescents [33]. Although children wanted their parents to speak with them about puberty and sex, the children reported *disgust* and *awkwardness* as the most common reaction towards discussions about sexual activity. Similarly, not all adults in the focus groups admitted to having these discussions with their children, and some described discussing sex as “very difficult" or “embarrassing.” These findings are also consistent with the experiences of other Hispanic parents of adolescents. Many lack confidence because they did not learn about sex or reproductive health from their own parents [34] because the topic was “taboo” in their culture [35]. Our study found that some parents felt that conversations surrounding sexual health were best left to the same-sex parent, a finding supported by the fact that Hispanic adolescents are more comfortable discussing these issues with the same gender parent [36].

Mirroring the responses of parents, children reported that their parents did not talk directly about sex. Instead, parents used *non-specific warning* to tell their children not to engage in sex (e.g., [referring to sex,] “Don’t do ‘it’ when you’re young”). Some parents felt that keeping communication brief would prevent creating more curiosity about sex in their child. A similar study conducted with a sample of predominately Cuban mothers found the ‘non-specific warning’ strategy to be one of the most-utilized methods among sex conversations with their teenage daughters [37].

When participants were asked about conversations around social media use, both child and parent respondents shared that their discussions centered around physical safety and privacy. Pre-adolescent children believed that their parents warned them about adults who had dangerous and predatory intent, and parents echoed similar fears and past conversations with their children. These findings correspond to prior evidence that Hispanic parents, among other minority parents, have higher levels of concern compared to White parents about their children’s safety on social media, especially when it comes to encountering strangers [38]. Because parents are typically focused on the physical safety dangers of social media, there is potential for future interventions to expand parent-child communication about other negative aspects of social media use including exposure to risk-taking behaviors. The authors found that Hispanic parents in this study were worried about the prospects of their pre-adolescent children liking members of the same sex, typically due to a conflict with their personal beliefs. One parent admitted that having a conversation with their child about the topic would require an “open mind” while another expressed unfavorable feelings even though she thought that parents ought to accept their children unconditionally. Ashcraft and Murray have also identified that attraction to members of the same sex is one of the challenging topics for the parents of adolescents and that parents be open-minded and “listen more than they speak” [39]. Additionally, Oerther illustrated how parents creatively came up with ways to maintain open communication with their children [40]. One way was to create a question box so that their pre-adolescent children could write questions they may not want to ask in-person. As a result, a parent was able to engage in a conversation with her female child who was exploring her feeling about attraction to a girl. Such an approach gave the parent time to think about how to approach what the child was experiencing. As a result, this parent was able to listen to the child, help the child process her feelings, foster the child in building the skills needed to think independently. This approach can be applied not only when exploring their attraction to the same sex or opposite sex persons but also regarding other areas of their identity (e.g., racial/ethnic identity). Thus, a parent who strives to be in tune with and cultivate a nurturing relationship with the child will positively contribute to supporting the child’s overall development and how the child should handle his/her exposure to risk-taking behaviors as they age.

The participants in this study, both parents and children, found the discussion of substances elicited less feelings of awkwardness or disgust than the discussion of sex. Any discomfort that children noted occurred when discussing illegal substances and when children were accused. Parents noted a discomfort with substances that were new to them, namely e-cigarettes. One parent noted that dealing with a vaping situation (the child held a vaping device, and the parent thought the child was vaping) with her child was ‘emotional’ as e-cigarettes and vaping were not common topics discussed when they were young. While there currently is a gap in the literature pertaining to Hispanic parents’ knowledge of the dangers of e-cigarettes, there are studies on general parental knowledge on this topic: Despite e-cigarette smoking being declared a national epidemic (25% of 15 to 24 year olds smoking e-cigarettes at least every once every 3 days), most parents are unaware of the dangers [41]. Ninety-six percent of parents know about JUUL, a popular brand of e-cigarette, but only 44% can visually identify it, and 73.5% report having no communication from their child’s school about the dangers of its use [42]. Thus, further focus on educating Hispanic parents and youth about e-cigarettes is important.

It is known that risk taking health behaviors that begin during the early adolescent stage are often carried into late adolescence and eventually adulthood [43]. Our study found that the pre-adolescent Hispanic children were aware of most of the presented risk-behavior topics (i.e., sexual activity and substance abuse) and had formed feelings about conversations on these topics that they have had with their parents, or the lack thereof. The authors of this study recommend future PCC interventions be conducted that target the Hispanic child at the pre-adolescent stage and test whether risk-behavior prevention is effective for this age group compared to early-to-late adolescents.

### Clinical implications

The generational gap between parents and children, particularly related to sexuality, puberty, and sex, is a pressing concern that arose from the focus groups. Further, parents expressed having a strong desire for more knowledge on effective communication with their children on these difficult topics. Pediatric healthcare providers (HCPs) could serve as an access point for resources regarding effective communication between parent and child.

Specifically, pediatric HCPs have the opportunity during well-child visits to observe the communication between parents and children. According to Bright Futures (a toolkit for pediatric care endorsed by the American Academy of Pediatrics), fostering parent-child relationship and communication is two of the areas pediatric HCPs need to address [44]. By assessing the effectiveness of this communication, the pediatric HCPs can facilitate and encourage safe and open dialogue. Moreover, the children in this study vocalized their preference for speaking about uncomfortable topics with their parents rather than speaking to others, including peers. Children may not be comfortable disclosing important health information, such as their sexual activity, menstruation, and puberty with their pediatric HCPs, so involving parents may be the key for children to obtain crucial information for health promotion purposes, which emphasizes the importance of ongoing parent-child communication. Therefore, reinforcing effective communication skills should be at the forefront of pediatric care.

Additionally, children discussed legal substances such as cigarettes and alcohol fairly comfortably in the focus groups. However, they were more reserved when discussing illegal drugs such as marijuana. On the other hand, parents grouped all substances, legal or not, in one category when discussing their feelings on this topic. This finding indicates that parents view all substances at the same risk level for their children. While the children in this study found it easier to differentiate between legal and illegal substances, parents should be more aware of these differences, especially in relation to prevalence, accessibility, and use among Hispanic pre-adolescents and adolescents. Therefore, pediatric HCPs should stay informed on the prevalence of substance use among pre-adolescents to educate parents and children on the consequences of participating in these risk-taking behaviors. From a clinical perspective, pediatric HCPs are at the forefront of addressing this knowledge gap and can provide up-to-date information on substances that are commonly used in their community. By staying informed on the trends of substance use, parents and pediatric HCPs have a better opportunity to identify signs of risk-taking behaviors related to substance use in pre-adolescents.

### Research implications

The results of this study indicate the need for interventions focused on equipping parents with effective communication skills. Promoting parent-child communication through research interventions may lead to a stronger relationship and greater trust in discussing uncomfortable topics. Importantly, identifying signs of potential risk-taking behaviors in children is essential. Therefore, interventions should continue to include both parents and children to maintain both perspectives of this dynamic.

Additionally, parents shared concern about their children’s behavior related to social media access and use. Further research on social media monitoring and mediation between parents and children is needed to gather insight regarding safety and appropriate conduct online.

### Limitations

This study has two limitations. One limitation is that the recruitment of study participants was gathered from the same community-based organization, where there is possibility that the children had received or were currently receiving other interventions. Another limitation is the disproportionate ratio between mothers and fathers (one father out of 24 participants) despite invitations to recruit fathers. There is a potential for parent-child communication to vary depending on the sex of the parent involved due to circumstances including but not limited to single-parent households, less involved fathers, or children feeling more comfortable discussing topics such as puberty with the parent that shares their gender. The overwhelming female majority in the parent focus groups leads to understanding parent-child communication mainly through the perspective of mothers. Therefore, any potential distinguishing patterns within parent-child communication that would be revealed through the perspective of fathers would not be noticed. Though the research team does not consider it to be a “limitation,” we acknowledge the demographic uniqueness of the focus group participants, Hispanic mothers (and one Hispanic father) from different countries of origin and residing in South Florida. The research team also acknowledges that this study was done in partnership with a private, community-based organization that operates an afterschool program. Therefore, when transferability is considered, such unique settings need to be considered.

## Conclusion

The results of the present study, combined with the existing literature, indicate that Hispanic pre-adolescent children may be at an increased risk for engaging in the risk-taking behaviors of substance use and high-risk sexual behaviors. Communication between parents, particularly mothers, and their pre-adolescent children is essential in decreasing these health risk behaviors. However, this communication may be hampered by the parent’s lack of knowledge or a disconnect between the information provided by the parent and the information that the pre-adolescent wishes to receive from the parent. More research needs to be conducted to determine the most effective ways to provide this information to parents so that parents can engage in meaningful conversations about risk reduction with their pre-adolescent children.

## References

[1] APA Dictionary of Psychology, https://dictionary.apa.org/ (accessed 30 August 2022).

[2] Babore A, Trumello C, Candelori C, et al. Depressive Symptoms, Self-Esteem and Perceived Parent–Child Relationship in Early Adolescence. *Front Psychol* 2016; 7: 1–7. 27445941 10.3389/fpsyg.2016.00982 PMC4923143

[3] Lansford JE, Dodge KA, Fontaine RG, et al. Peer Rejection, Affiliation with Deviant Peers, Delinquency, and Risky Sexual Behavior. *J Youth Adolesc* 2014; 43: 1742–1751. doi: 10.1007/s10964-014-0175-y 25150986 PMC4163526

[4] Lees B, Meredith LR, Kirkland AE, et al. Effect of alcohol use on the adolescent brain and behavior. *Pharmacol Biochem Behav* 2020; 192: 172906. doi: 10.1016/j.pbb.2020.172906 32179028 PMC7183385

[5] Evans D, Tawk R. The Relationship between Substance Abuse and Suicide among Adolescents. *Fla Public Health Rev*; 13, https://digitalcommons.unf.edu/fphr/vol13/iss1/8 (2019).

[6] Morris JL, Rushwan H. Adolescent sexual and reproductive health: The global challenges. *Int J Gynecol Obstet* 2015; 131: S40–S42. doi: 10.1016/j.ijgo.2015.02.006 26433504

[7] Kloess JA, Beech AR, Harkins L. Online Child Sexual Exploitation: Prevalence, Process, and Offender Characteristics. *Trauma Violence Abuse* 2014; 15: 126–139. doi: 10.1177/1524838013511543 24608540

[8] Vannucci A, Simpson EG, Gagnon S, et al. Social media use and risky behaviors in adolescents: A meta-analysis. *J Adolesc* 2020; 79: 258–274. doi: 10.1016/j.adolescence.2020.01.014 32018149

[9] Bucknell Bossen C, Kottasz R. Uses and gratifications sought by pre-adolescent and adolescent TikTok consumers. *Young Consum* 2020; 21: 463–478.

[10] Johnston LD, Miech RA, O’Malley PM, et al. *Monitoring the Future National Survey Results on Drug Use*, *1975–2019*: *Overview*, *Key Findings on Adolescent Drug Use*. Institute for Social Research, https://eric.ed.gov/?id=ED604018 (January 2020, accessed 31 August 2022).

[11] Miech R, Patrick ME, O’Malley PM, et al. What Are Kids Vaping? Results from a National Survey of U.S. Adolescents. *Tob Control* 2017; 26: 386–391. 27562412 10.1136/tobaccocontrol-2016-053014 PMC5326604

[12] Lantos H, Manlove J, Wildsmith E, et al. Parent-Teen Communication about Sexual and Reproductive Health: Cohort Differences by Race/Ethnicity and Nativity. *Int J Environ Res Public Health* 2019; 16: 833. doi: 10.3390/ijerph16050833 30866486 PMC6427285

[13] Romo DL, Garnett C, Younger AP, et al. Social Media Use and its Association with Sexual Risk and Parental Monitoring among a Primarily Hispanic Adolescent Population. *J Pediatr Adolesc Gynecol* 2017; 30: 466–473. doi: 10.1016/j.jpag.2017.02.004 28216129

[14] Rusby JC, Light JM, Crowley R, et al. Influence of parent–youth relationship, parental monitoring, and parent substance use on adolescent substance use onset. *J Fam Psychol* 2018; 32: 310–320. doi: 10.1037/fam0000350 29300096 PMC5920742

[15] Peterson I, Bhana A, Flisher AJ, et al. *Promoting mental health in scare-resource contexts*: *Emerging evidence and practice*. Cape Town, South Africa: Human Sciences Research Council, https://open.uct.ac.za/bitstream/handle/11427/2412/Promoting_Mental_Health.pdf?sequence=1&isAllowed=y#page=134 (2010).

[16] Kuntsche S, Kuntsche E. Parent-based interventions for preventing or reducing adolescent substance use—A systematic literature review. *Clin Psychol Rev* 2016; 45: 89–101. doi: 10.1016/j.cpr.2016.02.004 27111301

[17] Guilamo-Ramos V, Goldberg V, Lee JJ, et al. Latino Adolescent Reproductive and Sexual Health Behaviors and Outcomes: Research Informed Guidance for Agency-Based Practitioners. *Clin Soc Work J* 2012; 40: 144–156. doi: 10.1007/s10615-011-0355-0 23279981 PMC3532516

[18] Estrada Y, Lee TK, Huang S, et al. Parent-Centered Prevention of Risky Behaviors Among Hispanic Youths in Florida. *Am J Public Health* 2017; 107: 607–613. doi: 10.2105/AJPH.2017.303653 28207330 PMC5343711

[19] Widman L, Choukas-Bradley S, Noar SM, et al. Parent-Adolescent Sexual Communication and Adolescent Safer Sex Behavior: A Meta-Analysis. *JAMA Pediatr* 2016; 170: 52–61. doi: 10.1001/jamapediatrics.2015.2731 26524189 PMC4857605

[20] Prado G, Cordova D, Huang S, et al. The efficacy of Familias Unidas on drug and alcohol outcomes for Hispanic delinquent youth: Main effects and interaction effects by parental stress and social support. *Drug Alcohol Depend* 2012; 125: S18–S25. doi: 10.1016/j.drugalcdep.2012.06.011 22776441 PMC3435476

[21] Guilamo-Ramos V, Lee JJ, Kantor LM, et al. Potential for Using Online and Mobile Education with Parents and Adolescents to Impact Sexual and Reproductive Health. *Prev Sci* 2015; 16: 53–60. doi: 10.1007/s11121-014-0469-z 24522898

[22] Sandelowski M. Whatever happened to qualitative description? *Res Nurs Health* 2000; 23: 334–340. doi: 10.1002/1098-240x(200008)23:4&lt;334::aid-nur9&gt;3.0.co;2-g 10940958

[23] Sandelowski M. What’s in a name? Qualitative description revisited. *Res Nurs Health* 2010; 33: 77–84. doi: 10.1002/nur.20362 20014004

[24] Jamshed S. Qualitative research method-interviewing and observation. *J Basic Clin Pharm* 2014; 5: 87–88. doi: 10.4103/0976-0105.141942 25316987 PMC4194943

[25] Hsieh H-F, Shannon SE. Three Approaches to Qualitative Content Analysis. *Qual Health Res* 2005; 15: 1277–1288. doi: 10.1177/1049732305276687 16204405

[26] Graneheim UH, Lundman B. Qualitative content analysis in nursing research: concepts, procedures and measures to achieve trustworthiness. *Nurse Educ Today* 2004; 24: 105–112. doi: 10.1016/j.nedt.2003.10.001 14769454

[27] Creswell JW, Poth CN. Chapter 8: Data Analysis and Representation. In: Qualitative Inquiry & Research Design: Choosing Among Five Approaches. In: *Qualitative Inquiry and Research Design*: *Choosing Among Five Approaches*. Los Angeles: SAGE Publications, 2016.

[28] McHugh ML. Interrater reliability: the kappa statistic. *Biochem Medica* 2012; 22: 276–282. doi: 10.1016/j.jocd.2012.03.005 23092060 PMC3900052

[29] Polit DF, Beck CT. *Nursing research*: *generating and assessing evidence for nursing practice*. Tenth edition. Philadelphia: Wolters Kluwer Health, 2017.

[30] Lincoln YS, Guba EG. Naturalistic Inquiry. Newbury Park: SAGE Publications, https://us.sagepub.com/en-us/nam/naturalistic-inquiry/book842 (1985, accessed 30 August 2022).

[31] Denzin NK. *The Research Act*: *A Theoretical Introduction to Sociological Methods*. 2nd ed. New York, NY: McGraw-Hill, 1978.

[32] Patton MQ. Enhancing the quality and credibility of qualitative analysis. *Health Serv Res* 1999; 34: 1189–1208. 10591279 PMC1089059

[33] Eversole JS, Berglas NF, Deardorff J, et al. Source of Sex Information and Condom Use Intention Among Latino Adolescents. *Health Educ Behav* 2017; 44: 439–447. doi: 10.1177/1090198116671704 27899688

[34] Johnson-Motoyama M, Moses M, Kann TK, et al. Parent, Teacher, and School Stakeholder Perspectives on Adolescent Pregnancy Prevention Programming for Latino Youth. *J Prim Prev* 2016; 37: 513–525. doi: 10.1007/s10935-016-0447-2 27628931

[35] Alcalde MC, Quelopano AM. Latin American immigrant women and intergenerational sex education. *Sex Educ* 2013; 13: 291–304.

[36] Estrada-Martínez LM, Grossman JM, Richer AM. Sex behaviours and family sexuality communication among Hispanic adolescents. *Sex Educ* 2021; 21: 59–74. doi: 10.1080/14681811.2020.1749042 35814266 PMC9262336

[37] Matsuda Y, DeBastiani SD, Thalasinos RD, et al. Hispanic Mother-Daughter Communication About the Risks of Sex, Drugs, and Alcohol: Influences and the Strategies Mothers Use. *J Pediatr Nurs* 2021; 61: 325–330. doi: 10.1016/j.pedn.2021.08.026 34530374 PMC9117012

[38] Boyd D, Hargittai E. Connected and concerned: Variation in parents’ online safety concerns. *Policy Internet* 2013; 5: 245–269.

[39] Ashcraft AM, Murray PJ. Talking to Parents About Adolescent Sexuality. *Pediatr Clin North Am* 2017; 64: 305–320. doi: 10.1016/j.pcl.2016.11.002 28292447 PMC5517036

[40] Oerther S. Parenting Concerns: Uncovering Barriers and Identifying Ways to Support Pre-teen Flourishing. *West J Nurs Res* 2023; 45: 696–705. doi: 10.1177/01939459231180222 37288541

[41] Vallone DM, Bennett M, Xiao H, et al. Prevalence and correlates of JUUL use among a national sample of youth and young adults. *Tob Control* 2019; 28: 603–609. doi: 10.1136/tobaccocontrol-2018-054693 30377241

[42] Patel M, Czaplicki L, Perks SN, et al. Parents’ Awareness and Perceptions of JUUL and Other E-Cigarettes. *Am J Prev Med* 2019; 57: 695–699. doi: 10.1016/j.amepre.2019.06.012 31420121

[43] Brener ND, Kann L, Shanklin S, et al. Methodology of the Youth Risk Behavior Surveillance System—2013. 62(1), Centers for Disease Control and Prevention. 23446553

[44] Bright Futures—Implementation Tip Sheet—Practical Tips for Promoting Relational Health, https://downloads.aap.org/AAP/PDF/BF_RelationalHealth_Tipsheet.pdf (2022).

